# The ratios of dietary non-fibrous carbohydrate (NFC) to neutral detergent fiber (NDF) influence intestinal immunity of rabbits by regulating gut microbiota composition and metabolites

**DOI:** 10.3389/fmicb.2023.1146787

**Published:** 2023-04-20

**Authors:** Shuo Li, Tingting Liu, Kun Wang, Chong Li, Fengyang Wu, Xinyu Yang, Man Zhao, Baojiang Chen, Xiang Chen

**Affiliations:** ^1^College of Animal Science and Technology, Hebei Agricultural University, Baoding, China; ^2^College of Animal Science, Guizhou University, Guiyang, China; ^3^Institute of Cereal and Oil Crops, Hebei Academy of Agriculture and Forestry Sciences, Shijiazhuang, China; ^4^Key Laboratory for Feed Biotechnology of the Ministry of Agriculture and Rural Affairs, Institute of Feed Research, Chinese Academy of Agriculture Sciences, Beijing, China; ^5^Precision Livestock and Nutrition Laboratory, Teaching and Research Centre (TERRA), Gembloux Agro-Bio Tech, University of Liège, Gembloux, Belgium; ^6^College of Food Science and Technology, Hebei Agricultural University, Baoding, China

**Keywords:** NFC/NDF, rabbit, intestinal immunity, metabolites, volatile fatty acids, propionate, gut microbiota

## Abstract

Carbohydrate is the most common macronutrient consumed across all phases of the diet and acts as a potential regulator in modulating the gut microbiota in animals. However, the influences of dietary non-fibrous carbohydrate (NFC) to neutral detergent fiber (NDF) in different ratios on gut microbiota, metabolites, intestinal immunity, and growth performance have not been fully explored. A total of 135 healthy weaned rabbits (45.1 ± 0.7 d of age) with an average body weight of 1.08 ± 0.07 kg were randomly divided into five groups. Under the same other nutrient levels, rabbits were fed diets with NFC/NDF ratios of 0.7 (T1), 1.0 (T2), 1.3 (T3), 1.6 (T4), and 1.9 (T5). During the 28-day experiment, T3 rabbits showed the highest final body weight and the lowest feed-to-weight ratio than T5 rabbits (*P* < 0.05) but no significant difference with T1 or T2 rabbits. The expression of cecal pro-inflammatory factors IL-1β and TNF-α was increased in the T4 and T5 than in those of other groups (*P* < 0.05). Conversely, the tight junction proteins (ZO-1, Claudin-1, and Occludin) were decreased to varying degrees in the T4 and T5 groups. The pH value in the cecal digesta of T5 rabbits was lower than that of T1, T2, and T3 (*P* < 0.05), while the concentration of volatile fatty acids and propionate was higher than those of T1, T2, and T3 rabbits (*P* < 0.05). In terms of gut microbiota, at the phylum level, the relative burden of Firmicutes and Actinobacteria in T2 rabbits was the highest (*P* < 0.05), and the relative burden of Proteobacteria in T5 rabbits was higher than that of other groups (*P* < 0.05). At the genus level, the relative burden of *Ruminococcus* was higher in T2 and T3 rabbits than that of other groups, and T5 rabbits have the lowest relative burden of *Ruminococcus*. Combination analysis showed that cecal metabolites were positively associated with fermentation-related phenotypes and the burden of Firmicutes (*P* < 0.05). In conclusion, different dietary NFC/NDF ratios can affect the intestinal immune response and growth performance of rabbits, and there was a positive effect when dietary NFC/NDF = 1.0–1.3.

## 1. Introduction

Intestinal microorganisms can reduce the pH of the intestinal content by degrading dietary fiber, which promotes the colonization of the acidophilic flora in the intestine, such as *Lactobacillus, Bifidobacterium, Bacteroides*, and *Faecalibacterium* (Holscher, 2017; Makki et al., 2018). Previous studies have reported that different types of fibers have a significant effect on the gut microbiome diversity in animals, and the number of fiber-degrading bacteria in the cecum increases when the fiber content of the diet is increased from 11 to 17% (Jin et al., 2018). Increasing dietary fiber can reduce the residence time of the digesta in the cecum and inhibits the growth of pathogenic microorganisms (Gidenne, 2015). Dietary fiber affects the growth and immune performance of rabbits by altering the structure of the gut microbiome and then influences intestinal immunity (Eberl, 2010; Zheng et al., 2020; Ye et al., 2021; Xue et al., 2022). It was found that dietary soluble fiber may protect the intestinal mucosa by limiting the penetration of acetylsalicylic acid into epithelial cells. Furthermore, volatile fatty acids (VFAs) produced by fermentative degradation of dietary fiber in the intestine can resist potentially infectious agents *in vivo* (Che et al., 2019; Shang et al., 2020; Guan et al., 2021). When the integrity of the intestinal epithelial barrier is compromised, potentially harmful macromolecules can easily pass through the intestinal epithelial barrier into the organism and result in autoimmune diseases (Iliev and Cadwell, 2021).

Neutral detergent fiber (NDF) can be degraded by intestinal microorganisms into VFA to supply energy for the organism. Studies have shown that dietary NDF levels could affect cecal-digesta pH and VFA concentration in rabbits, and the amount of anaerobic fungal increased with the increasing dietary NDF level, VFA produced by anaerobic fungal fermentation can promote the expression of intestinal tight junction proteins to avoid inflammatory reactions in the organism, which is beneficial to intestinal health (Zhu et al., 2017; Goulart et al., 2020). In addition, it has been reported that an increasing dietary NDF can improve the intestinal development and immune defense of rabbits (Zhu et al., 2017; Wu et al., 2019). However, excessive NDF can lead to a decrease in apparent nitrogen digestibility, which affects the growth performance of rabbits (Zhu et al., 2020).

Non-fibrous carbohydrates (NFCs) in diets are important components of carbohydrates that can provide a large amount of blood glucose to animals with negative energy balance (Hall, 2003). As a result, it was generally accepted that diets with high NFC concentration are more conducive to promoting the growth and development of the organism (Xue et al., 2019; Hernández-Castellano et al., 2020; Khattab et al., 2021). However, excessive NFC concentration can increase the odds of developing rumen acidosis and diarrhea, which also have a negative impact on growth performance (Xue et al., 2019; Li et al., 2022).

Recent studies have shown that an unbalanced NFC/NDF ratio in the diet can affect the disturbances in the rumen environment and a reduction in bacterial diversity of ruminants, which may eventually result in a reduction of growth performance (Pu et al., 2020; Chen et al., 2022). However, there are a very limited number of studies on monogastric animals. As a representative model system, rabbits have been extensively used as experimental models in biomedical research. This experiment was conducted to investigate the effects of different dietary NFC/NDF on gut microbiota, metabolites, and immune homeostasis in rabbits, and the result will also provide a scientific basis for determining the appropriate dietary NFC/NDF ratio of rabbits. We hypothesized that dietary NFC/NDF at a relatively low ratio (<1.4) had a positive effect on the growth and immune performance of rabbits, whereas a relatively high ratio (>1.4) had a negative effect.

## 2. Materials and methods

### 2.1. Animals, management, and experimental diets

A total of 135 healthy weaned New Zealand White rabbits (45.1 ± 0.7 d of age, average weight 1.08 ± 0.07 kg) were randomly divided into five groups, respectively. Each group was further allocated into 27 replicates, with a rabbit per replicate. Under the condition of the same other nutrient levels, rabbits were fed diets with NFC/NDF of 0.7 (T1), 1.0 (T2), 1.3 (T3), 1.6 (T4), and 1.9 (T5). The experiment period is 28 days. During the experiment, all rabbits were allowed to eat and drink freely. The rabbit dormitory was ensured with normal light and ventilation. The diet was formulated according to the nutritional requirements of the National Research Council (1977), using corn, bran, soybean meal, alfalfa meal, and oat meal as raw materials, formulated into pellet feed with a diameter × length of 4 × 10 mm. The feed composition is provided in Supplementary Table 1.

### 2.2. Sampling

In order to evaluate the growth performance parameters, body weight (BW) and feed consumption were measured; average daily gain (ADG), average daily feed intake (ADFI), and the feed conversion ratio (FCR) were calculated (g feed/g gain) for all phases. The number of dead rabbits with diarrhea was recorded during the experiment, and the mortality and diarrhea rates were calculated *via* the following formulas. Mortality (%) = (the number of dead rabbits/total number of experimental rabbits) × 100%. Diarrhea (%) = (the number of rabbits with diarrhea/total number of experimental rabbits) × 100%. On the 28th day of the trial, six rabbits from each replicate were randomly selected; blood samples were taken (2.5 mL) from the marginal vein of the ear using an anticoagulant-free vacuum test tube (5 mL) and immediately transported for analysis of routine hemogram with an auto hematology analyzer (Mindray BC-2800, Shenzhen, China). Afterward, the rabbits were euthanized according to AVMA (Association, 2001). The cecal tissue and content were immediately frozen in liquid nitrogen at −80°C till analyzed for mRNA, volatile fatty acid (VFA), and gut microbiota.

The mRNA expression of genes related to gut immunity interleukin-1β (IL-1β), tumor necrosis factor-alpha (TNF-α), Claudin-1, Occludin, zonula occludens (ZO-1), and GAPDH in cecum tissues was measured by RT-qPCR. Primers were designed using Premier 5.0 software (Table 1), and GAPDH was used to normalize the expression of the targeted genes. Procedure: (1) The total RNA from the intestinal mucosa was isolated by TRI-zol reagent (TIANGEN, Beijing, China). (2) The total RNA was determined from OD 260/280. (3) Then, the total RNA was reversely transcribed into cDNA using the prime script of Fast Quant RT Kit (TIANGEN, Beijing, China), and qPCR was conducted. (4) The mRNA level of the relative gene was calculated using the 2^−ΔΔCt^ method. Each sample was analyzed in triplicate and the results are reported as the geometric mean of the three results.

**Table 1:** RT-PCR primer sequences.

**Genes**	**Accession number**	**Primer sequence (5^′^-3^′^) Sense/antisense forward primer**	**Production length (bp)**
Claudin-1	NM_001089316.1	Forward: CGTGCCTTGATGGTGATTGG	112
		Reverse: TCCGCATCTTTTGCTCCTCA	
Occludin	XM_017344772.1	Forward: TCCGACTTCGTGGAGAGAGT	126
		Reverse: AAGCTCATGAACCACCTCGG	
ZO-1	XM_017348360.1	Forward: AGAGGATTTGTCAGCCCAGC	125
		Reverse: TTTCTCTGGCAACATCGGCT	
IL-1β	NM_001082201.1	Forward: TTTGAGTCTGCCCAGTTCCC	77
		Reverse: TTGTTTCCCAGGAAGACGGG	
TNF-α	NM_001082263.1	Forward: CTCAGGAGGAAGAGTCCCCAA	115
		Reverse: GCTACTACGTGGGCTAGAGG	
GAPDH	NM_001082253.1	Forward: CGAGACACGATGGTGAAGGT	165
		Reverse: GCCGTGGGTGGAATCATACT	

The VFA of the cecal content was measured by the Agilent 7890A gas chromatograph (Agilent Technologies, Santa Clara, United States), according to the integration parameters and calibration curve. The pH value was detected potentiometrically using an automated pH analyzer (PHSJ-3F, INESA scientific instrument Co. Ltd, Shanghai, China). The NH_3_-N of the cecal content was measured by the phenol-sodium hypochlorite colorimetric method. A measure of 1 mL of sample solution or standard solution was added to 4 mL of HCl (0.2 mol/L) solution and then mixed well. After that, 0.4 mL of the mixture was added to 5 mL of phenol reagent, and 4 mL of sodium hypochlorite was added and mixed well. The sample was water bath-heated at 60°C for 10 min, and after cooling, the absorbance was measured at 545 nm wavelength (Weatherburn, 1967).

### 2.3. Cecal content DNA extraction, 16S rRNA amplification, and sequencing

A total of six cecal content samples from each group were randomly selected and used for the analysis of the gut microbiota. The workflow included DNA extraction, quality inspection, database sequencing, and 16S rRNA gene sequencing. Detailed methods are available in Supplementary material 2.

### 2.4. Statistical analysis

The results obtained were analyzed for power analysis in the G^*^Power package (version 3.1.4.), to ensure adequate power for the analysis. Afterward, experimental data were tested for normality by using the Shapiro–Wilk test of normality and for homogeneity of variances by using Levene's test. An overall test for treatment effect was first performed with a one-way analysis of variance (ANOVA). Linear and quadratic trends were conducted using orthogonal polynomial coefficients (Social Sciences 19.0 software, SPSS Inc., Chicago, IL, United States). The indexes were expressed as means with standard error of the mean (SEM) and significant main effect. *P* < 0.05 was considered a significant difference. GraphPad-Prism version 7.0 (San-Diego, CA, United States) and heatmap illustrator (HemI, version 1.0.3.7) software were used to draw the graphs and heatmaps, respectively.

## 3. Results

### 3.1. Effects of dietary NFC/NDF on growth performance

As presented in Table 2, the BW and ADG of rabbits in T5 were lower than the other groups (*P* < 0.05), and the ADFI of rabbits in T1, T2, and T3 was significantly higher than that in T4 and T5. Polynomial contrast analysis showed that the level of BW (*P*_lin_ < *0.001, P*_quad_ < 0.001), ADG (*P*_lin_ < 0.001, *P*_quad_ < 0.001), and ADFI (*P*_lin_ < 0.001, *P*_quad_ < 0.001) displayed linear and quadratic patterns of decrease with increasing dietary NFC/NDF inclusion level, with T1, T2, T3, and T4 being higher than T5. However, the F/G ratio in T5 rabbits was higher than that in the other groups (*P* < 0.05, *P*_lin_ < 0.001, and *P*_quad_ < 0.001), and the F/G ratio of rabbits in T3 was slightly lower than that in T1, T2, and T4 (*P* > 0.05). No individuals in the T1, T2, and T3 groups were found to have diarrhea or death. However, the diarrhea rates in the T4 and T5 groups were 18.52 and 59.26%, respectively, with the death rates being 7.41 and 14.81%, respectively.

**Table 2:** Effects of different ratios of dietary NFC/NDF on growth performance.

**Items**	**Treatment**					**SEM**	**Statistics**		
	**T1**	**T2**	**T3**	**T4**	**T5**		*P*_anova_	*P*_linear_	*P*_quadratic_
BW, g	2,505.95^ab^	2,499.06^ab^	2,563.38^a^	2,372.13^b^	2,076.06^c^	29.584	<0.001	<0.001	<0.001
ADG, g/day/rabbit	54.42^ab^	54.08^ab^	55.53^a^	49.11^b^	38.63^c^	1.065	<0.001	<0.001	<0.001
ADFI, g/day/rabbit	193.79^a^	179.31^a^	181.49^a^	157.04^b^	154.59^b^	2.724	<0.001	<0.001	<0.001
F/G ratio	3.57^b^	3.34^b^	3.28^b^	3.24^b^	4.25^a^	0.068	0.002	0.008	<0.001
Diarrhea rate, %	0	0	0	18.52	59.26	–	–	–	–
Mortality, %	0	0	0	7.41	14.81	–	–	–	–

BW, body weight; ADG, average daily gain; ADFI, average daily feed intake; F/G, feed/gain.

^a, b, c^Different superscript letters within a row indicate significant differences at *P* < 0.05. T1, NFC/NDF = 0.7; T2, NFC/NDF = 1.0; T3, NFC/NDF = 1.3; T4, NFC/NDF = 1.6; T5, NFC/NDF = 1.9.

### 3.2. Effects of dietary NFC/NDF on blood parameters

**Figure 1:**
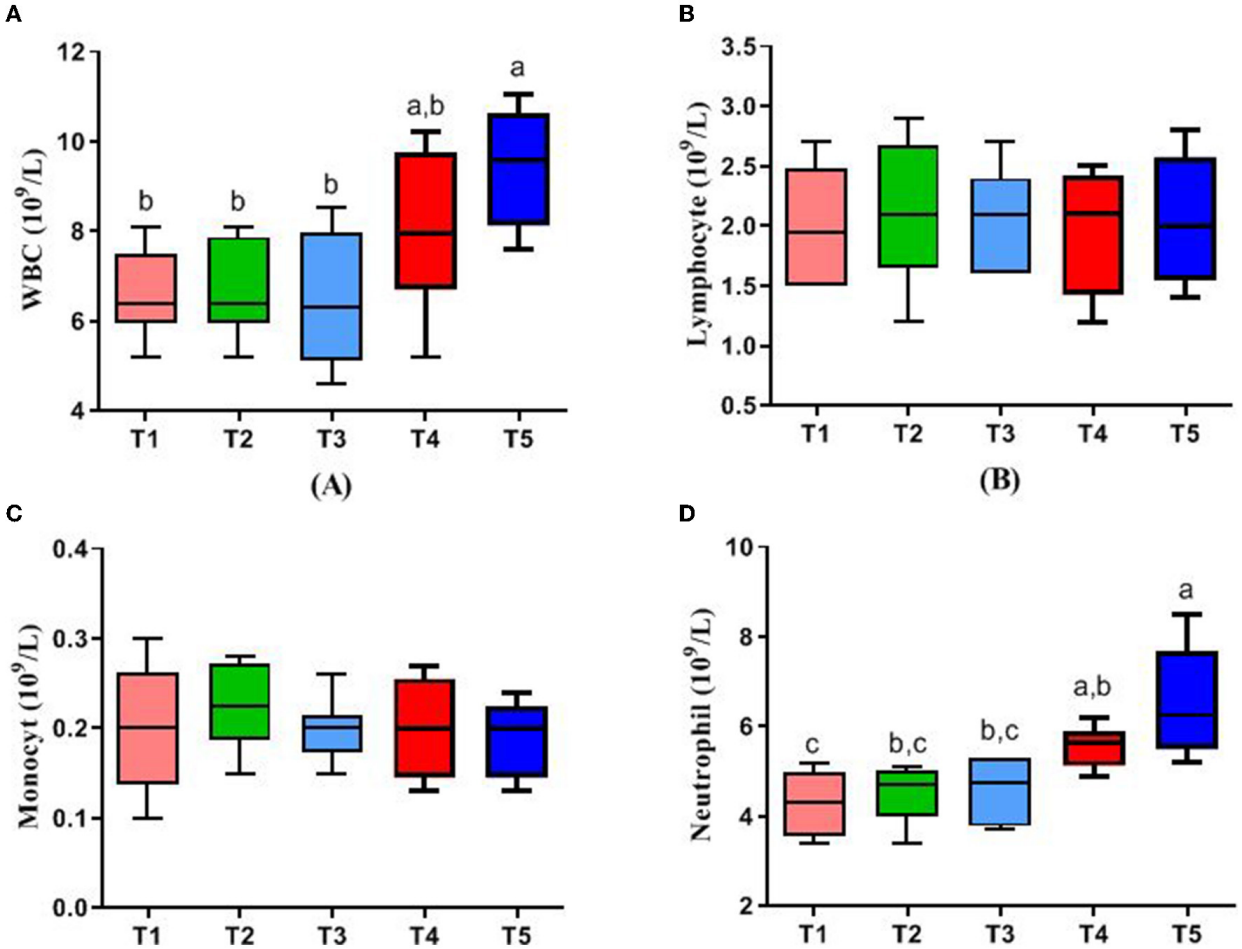
Effects of different ratios of dietary NFC/NDF on blood parameters. Data are indicated as means ± SEM (*n* = 6). **(A)** The impact on the number of white blood cells (WBC). **(B)** The impact on the number of lymphocytes. **(C)** The impact on the number of monocytes. **(D)** The impact on the number of neutrophils. T1, NFC/NDF = 0.7; T2, NFC/NDF = 1.0; T3, NFC/NDF = 1.3; T4, NFC/NDF = 1.6; T5, NFC/NDF = 1.9. *a, b*, and *c* Values, for the same parameter, with different superscripts are significantly different (*P* < 0.05).

Figure 1 represents the blood parameters of rabbits. WBC and neutrophil in the blood of rabbits in the T5 group were higher than those in T1, T2, and T3 (*P* < 0.05), while there were no significant differences in lymphocyte and monocyte in all groups (*P* > 0.05).

### 3.3. Effects of dietary NFC/NDF on the expression of genes related to intestinal immunity

**Figure 2:**
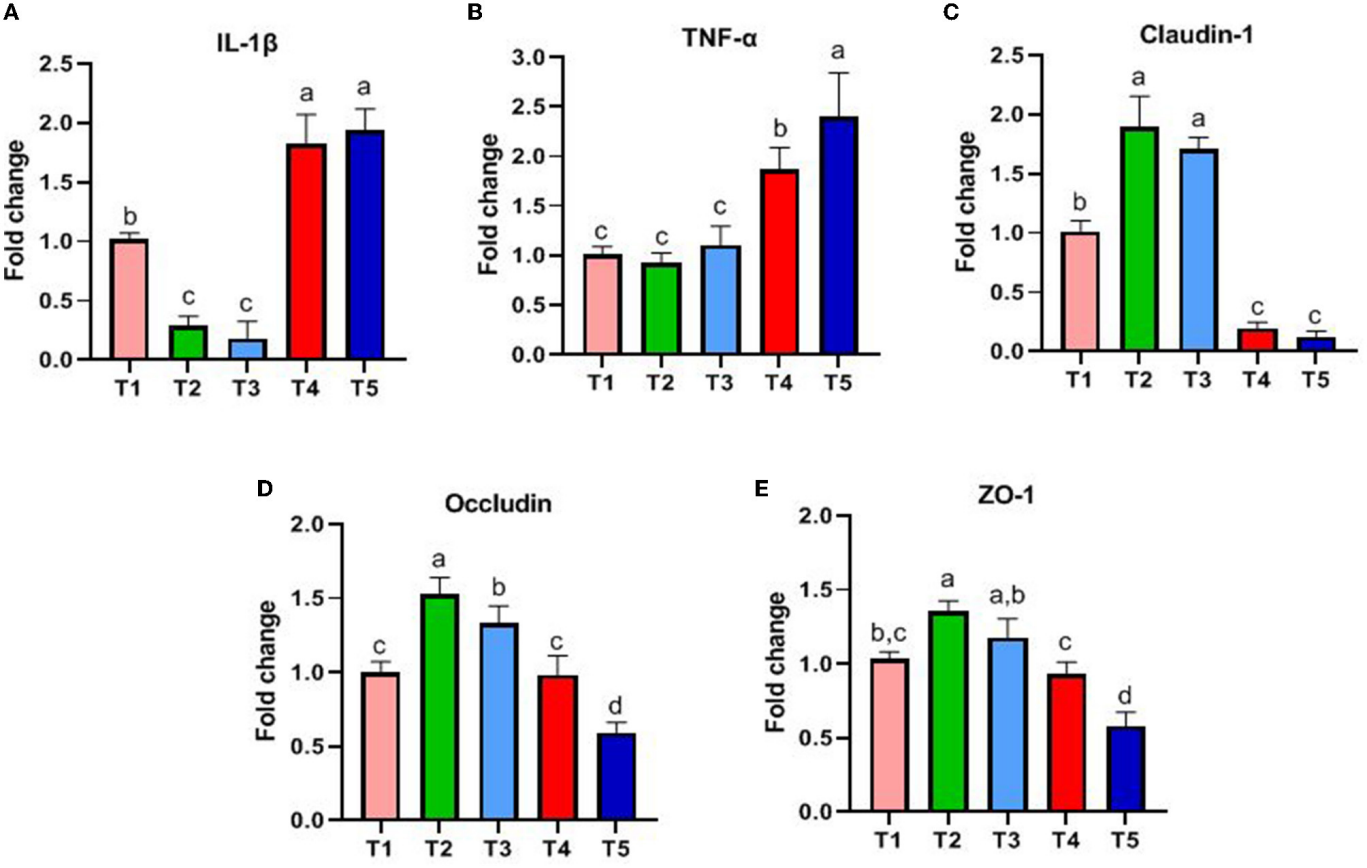
Effects of different ratios of dietary NFC/NDF on the expression of genes related to gut immunity. Data are indicated as means ± SEM (*n* = 6). **(A)** The impact on the expression of IL-1β. **(B)** The impact on the expression of TNF-α. **(C)** The impact on the expression of Claudin-1. **(D)** The impact on the expression of Occludin. **(E)** The impact on the expression of ZO-1. T1, NFC/NDF = 0.7; T2, NFC/NDF = 1.0; T3, NFC/NDF = 1.3; T4, NFC/NDF = 1.6; T5, NFC/NDF = 1.9. a, b, and c values for each intestinal segment with different superscripts are significantly different (*P* < 0.05).

The results are shown in Figure 2. The genes associated with innate intestinal immunity, and the expression of IL-1β and TNF-α in T4 and T5 rabbits was significantly higher than that in T1, T2, and T3 (*P* < 0.05, Figures 2A, B). The genes associated with the intestinal barrier and the expression of Claudin-1 and Occludin in T2 and T3 rabbits were higher than that in other groups (*P* < 0.05, Figures 2C, D). ZO-1 in T2 rabbits was higher than that in T1, T4, and T5 (*P* < 0.05, Figure 2E).

### 3.4. Effects of dietary NFC/NDF on cecal pH and metabolites

As shown in Table 3, we can conclude that the pH of the cecal content of T5 rabbits was lower than that of T1, T2, and T3 (*P* < 0.05). The pH decreased in a linear (*P*_lin_ = 0.032) and a quadratic (*P*_quad_ = 0.005) fashion with increasing dietary NFC/NDF inclusion levels with T1, T2, and T3 being higher than T4 and T5. The level of VFA in T5 rabbits was higher than that in T1 and T2 (*P* < 0.05). With the increase in dietary NFC/NDF, the concentration of acetate decreased, which was highest in T1 and T2 rabbits than in other groups, especially compared with T5 (*P* < 0.05). However, the concentration of propionate in T5 rabbits was higher than that in T1, T2, and T3 (*P* < 0.05). Polynomial contrast analysis showed that the level of VFA (*P*_quad_ = 0.013) and propionate (*P*_quad_ < 0.001) displayed a quadratic pattern of increase with increasing dietary NFC/NDF inclusion level. But the expression of acetate displayed quadratic (*P*_quad_ = 0.014) and linear (*P*_lin_ = 0.005) patterns of decrease. Among these five groups, no significant difference was observed in NH_3_-N and butyrate (*P* > 0.05).

**Table 3:** Effects of different ratios of dietary NFC/NDF on cecal pH and metabolites.

**Items**	**Treatment**					**SEM**	**Statistics**		
	**T1**	**T2**	**T3**	**T4**	**T5**		*P*_anova_	*P*_linear_	*P*_quadratic_
pH	6.31^abc^	6.52^a^	6.45^ab^	6.24^bc^	6.11^c^	0.046	0.015	0.032	0.005
NH_3_-N, mg/dL	14.29	12.83	13.33	12.77	12.06	0.508	0.253	0.216	0.473
VFA, mmol/L	40.32^b^	39.45^b^	41.10^ab^	42.15^ab^	44.39^a^	0.609	0.010	0.008	0.013
Acetate, mmol/L	25.70^a^	26.36^a^	24.75^ab^	23.14^ab^	21.90^b^	0.584	0.008	0.005	0.014
Propionate, mmol/L	8.94^b^	9.27^b^	8.91^b^	11.02^a^	11.54^a^	0.301	<0.001	<0.001	<0.001
Butyrate, mmol/L	4.68	4.79	4.52	4.53	4.46	0.204	0.663	0.634	0.895

^a, b, c^Different superscript letters within a row indicate significant differences at *P* < 0.05. T1, NFC/NDF = 0.7; T2, NFC/NDF = 1.0; T3, NFC/NDF = 1.3; T4, NFC/NDF = 1.6; T5, NFC/NDF = 1.9.

### 3.5. Effects of dietary NFC/NDF on cecal microbial diversity

**Figure 3:**
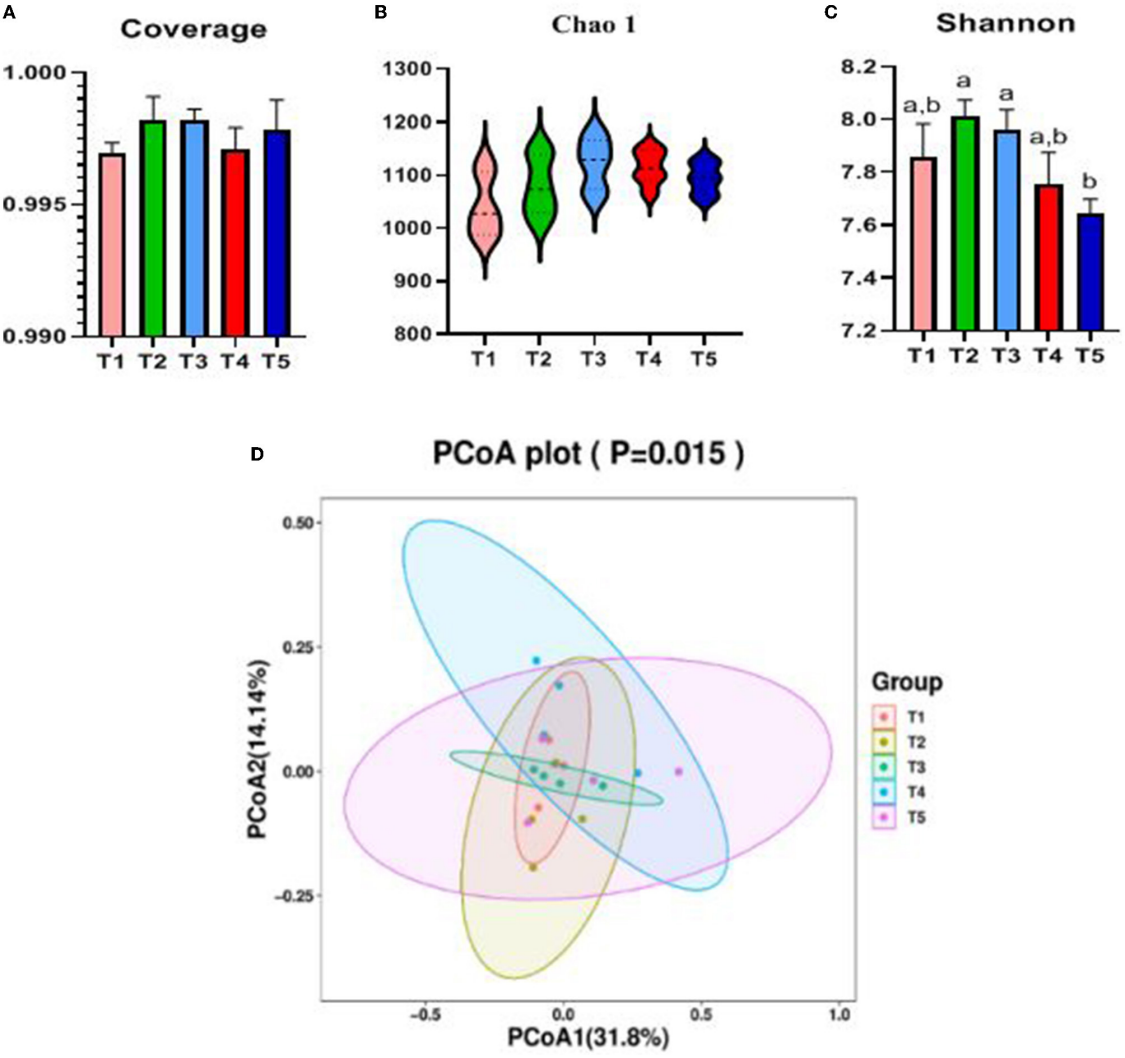
Effects of different ratios of dietary NFC/NDF on cecal microbial diversity. Data are indicated as means ± SEM (*n* = 6). **(A)** Coverage index of OUT level. **(B)** Chao1 index of OUT level. **(C)** Shannon index of OUT level. **(D)** β-diversity was estimated by the PCoA on the OUT level, respectively. T1, NFC/NDF = 0.7; T2, NFC/NDF = 1.0; T3, NFC/NDF = 1.3; T4, NFC/NDF = 1.6; T5, NFC/NDF = 1.9. a, b, and c Values for each intestinal segment with different superscripts are significantly different (*P* < 0.05).

The Alpha diversity of the cecal microbiota of rabbits was influenced by dietary NFC/NDF (Figures 3A–C). Coverage value and Chao1 microbial diversity indices were not significantly altered by dietary NFC/NDF. In terms of species diversity, Shannon in T5 rabbits was lower than that in T2 and T3 (*P* < 0.05). The cecal microbiota samples from T1, T2, and T3 were clustered together and clearly separated from the cecal microbiota of T4 and T5 rabbits, as shown in Figure 3D.

### 3.6. Effects of dietary NFC/NDF on cecal microbial composition

#### 3.6.1. Phylum level

**Figure 4:**
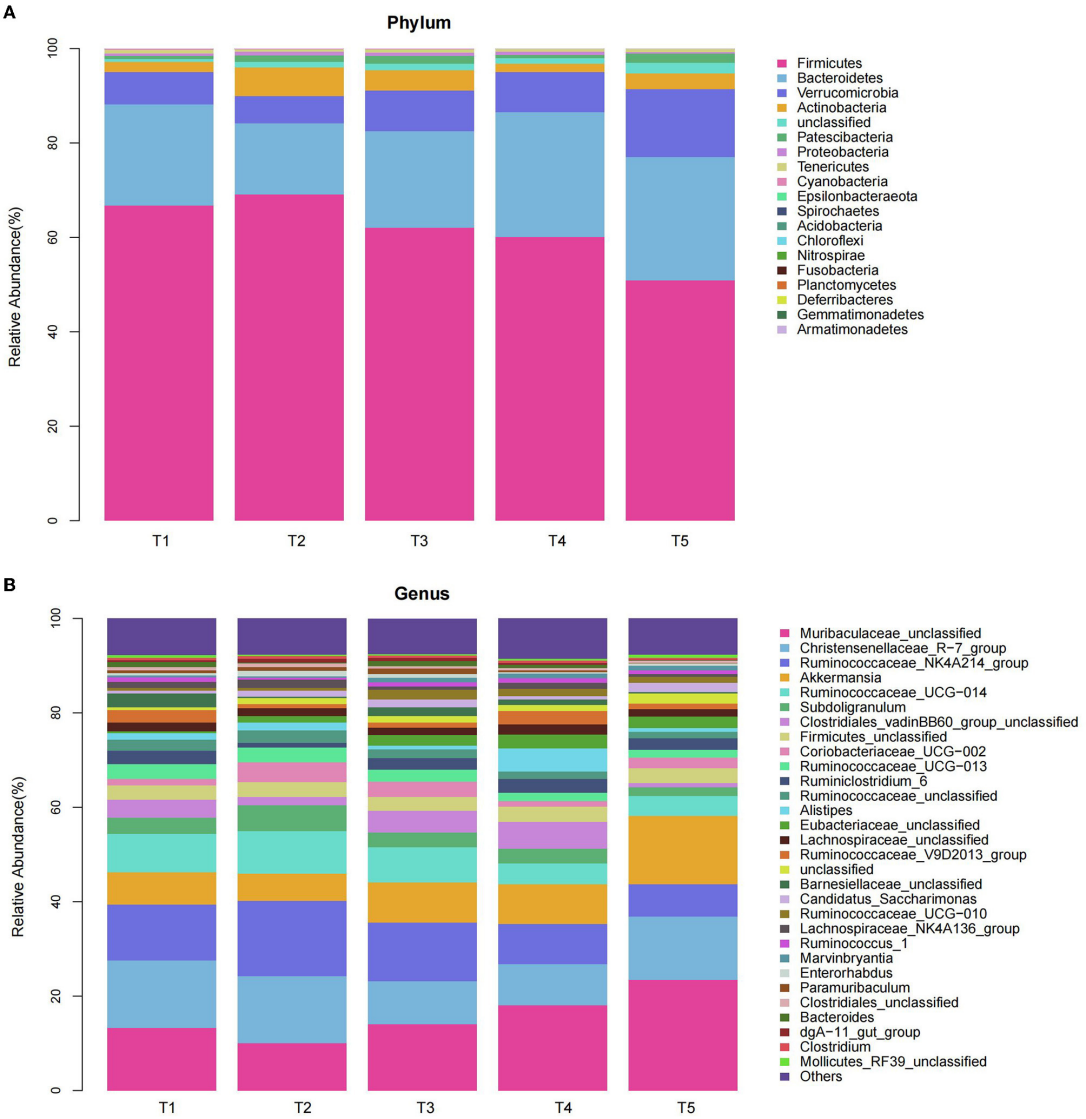
Effects of different ratios of dietary NFC/NDF on the relative abundances of bacterial Phylum **(A)** and Genus **(B)** in the cecal microbiota of rabbits. Data are indicated as means ± SEM (*n* = 6). T1, NFC/NDF = 0.7; T2, NFC/NDF = 1.0; T3, NFC/NDF = 1.3; T4, NFC/NDF = 1.6; T5, NFC/NDF = 1.9.

We compared the microbial communities at the phylum level. The predominant phyla were Firmicutes, Bacteroidetes, Verrucomicrobia, and Actinobacteria accounted for 97.14, 96.01, 95.42, 96.77, and 94.84% for T1, T2, T3, T4, and T5 groups, respectively (Figure 4A, Table 4). Compared with the T2 rabbits, dietary NFC/NDF changed the relative burden of Firmicutes in the T1 (−3.36%; *P* > 0.05), T3 (-10.11%; *P* > 0.05), T4 (−12.90%; *P* > 0.05), and T5 (−26.32%; *P* < 0.01) groups. The relative burden of Bacteroidetes differed (*P* = 0.003) between the experimental treatments, displaying linear (*P*_*lin*_ = 0.011) and quadratic (*P*_quad_ = 0.014) patterns of increase with increasing dietary NFC/NDF level, and the relative burden of Bacteroidetes in T2 rabbits was lower than that in T4 and T5 (*P* < 0.01) groups. The relative burden of Verrucomicrobia in T5 rabbits was higher than that in other groups (*P* < 0.01, *P*_lin_ < 0.001, and *P*_quad_ < 0.001). Compared with the T2 rabbits, dietary NFC/NDF changed the relative burden of Actinobacteria in the T1 (−64.11%; *P* < 0.01), T3 (−28.05%; *P* < 0.01), T4 (−70.95%; *P* < 0.01), and T5 (−43.57%; *P* < 0.01) groups. However, the relative burden of Proteobacteria increased in a linear (*P*_lin_ < 0.001) and a quadratic (*P*_quad_ < 0.001) fashion with increasing dietary NFC/NDF inclusion levels with T4 and T5 being higher than that in T1, T2, and T3 groups. The relative burdens of Patescibacteria, Tenericutes, Cyanobacteria, and Epsilonbacteraeota were all below 1%, and there were significant differences between all groups (*P* < 0.05).

**Table 4:** Effects of different ratios of dietary NFC/NDF on the distribution of cecal microflora in rabbits at the phylum level %.

**Items**	**Treatment**					**SEM**	**Statistics**		
	**T1**	**T2**	**T3**	**T4**	**T5**		*P*_anova_	*P*_linear_	*P*_quadratic_
Firmicutes	66.75^a^	69.07^a^	62.09^a^	60.16^ab^	50.89^b^	2.000	0.002	0.002	0.003
Bacteroidetes	21.44^a^	15.14^b^	20.43^ab^	26.36^a^	26.13^a^	1.203	0.003	0.011	0.014
Verrucomicrobia	6.80^b^	5.81^b^	8.59^b^	8.51^b^	14.44^a^	0.812	<0.001	<0.001	<0.001
Actinobacteria	2.15^c^	5.99^a^	4.31^b^	1.74^c^	3.38^b^	0.384	<0.001	0.524	0.217
Patescibacteria	0.61^c^	1.30^b^	1.66^ab^	0.65^c^	2.00^a^	0.135	<0.001	0.021	0.074
Proteobacteria	0.42^cd^	0.34^d^	0.54^c^	0.78^b^	0.93^a^	0.058	<0.001	<0.001	<0.001
Tenericutes	0.81^a^	0.45^c^	0.67^ab^	0.52^bc^	0.60^bc^	0.035	0.046	0.175	0.079
Cyanobacteria	0.13^a^	0.12^ab^	0.10^ab^	0.65^b^	0.09^ab^	0.009	0.025	0.020	0.044
Epsilonbacteraeota	0.035^a^	0.035^a^	0.001^b^	0.003^b^	0.003^b^	0.004	0.001	<0.001	<0.001

^a, b, c^Different superscript letters within a row indicate significant differences at *P* < 0.05. T1, NFC/NDF = 0.7; T2, NFC/NDF = 1.0; T3, NFC/NDF = 1.3; T4, NFC/NDF = 1.6; T5, NFC/NDF = 1.9.

#### 3.6.2. Genus level

We analyzed the microbial communities with a relative burden of >1% at the genus level (Figure 4B, Table 5). It was shown that the relative burdens of the *Ruminococcus_NK4A214_group, Ruminococcus_UCG-014*, and *Ruminococcus_UCG-013* in Firmicutes of T2 and T3 rabbits were higher than those of T4 and T5 (*P* < 0.05). In addition, polynomial contrast analysis showed that the level of the relative burden of *Ruminococcus_NK4A214_group* (*P*_quad_ < 0.001), *Ruminococcus_UCG-014* (*P*_quad_ < 0.001), *Ruminococcus_UCG-013* (*P*_quad_ < 0.002), and *Ruminococcaceae_unclassified* (*P*_quad_ < 0.001) displayed a quadratic pattern of decrease with increasing dietary NFC/NDF inclusion level. Compared with the T2 rabbits, dietary NFC/NDF changed the relative burden of *Coriobacteriaceae_UCG-002* in the T1 (−66.36%; *P* < 0.01), T3 (−25.00%; *P* < 0.01), T4 (−72.17%; *P* < 0.01), and T5 (−45.75%; *P* < 0.01) groups. Increasing dietary NFC/NDF inclusion level resulted in a quadratic pattern of increase for the relative burden of *Muribaculaceae_unclassified* (*P*_quad_ < 0.001) and *Akkermansia* (*P*_quad_ < 0.001) and resulted in a linear pattern of increase for the relative burden of *Candidatus_Saccharimonas* (*P*_lin_ = 0.024). Meanwhile, these three microbial at T5 were higher than other groups (*P* < 0.05). In addition, the relative burden of the *Subdoligranulum* differed (*P* < 0.001) between the experimental treatments, displaying linear (*P*_lin_ = 0.006) and quadratic (*P*_quad_ = 0.002) patterns of decrease with increasing dietary NFC/NDF levels. Meanwhile, the linear and quadratic patterns of decrease in the relative burden of *Lachnospiraceae_NK4A136_group* (*P*_lin_ = 0.010) with increasing dietary NFC/NDF level were noted. Furthermore, both the relative burden of *Subdoligranulum* and *Lachnospiraceae_NK4A136_group* reached the highest at T2 and the lowest at T5.

**Table 5:** Effects of different ratios of dietary NFC/NDF on the distribution of cecal microflora in rabbits at the genus level %.

**Items**	**Treatment**					**SEM**	**Statistics**		
	**T1**	**T2**	**T3**	**T4**	**T5**		*P*_anova_	*P*_linear_	*P*_quadratic_
*Ruminococcaceae_NK4A214_group*	11.93^b^	15.95^a^	12.42^b^	8.51^c^	6.85^c^	0.809	<0.001	<0.001	<0.001
*Ruminococcaceae_UCG-014*	8.17^ab^	8.98^a^	7.38^b^	4.31^c^	4.27^c^	0.477	<0.001	<0.001	<0.001
*Ruminococcaceae_UCG-013*	3.10^a^	3.09^a^	2.62^ab^	1.73^b^	1.57^b^	0.203	0.001	0.001	0.002
*Coriobacteriaceae_UCG-002*	1.40^d^	4.24^a^	3.18^b^	1.18^d^	2.30^c^	0.279	<0.001	0.541	0.141
*Muribaculaceae_unclassified*	13.26^c^	10.04^d^	14.05^c^	18.08^b^	23.40^a^	1.135	<0.001	<0.001	<0.001
*Akkermansia*	6.80^c^	5.81^c^	8.59^b^	8.51^b^	14.44^a^	0.719	<0.001	<0.001	<0.001
*Candidatus_Saccharimonas*	0.60^c^	1.32^b^	1.63^ab^	0.65^c^	2.00^a^	0.137	<0.001	0.024	0.085
*Ruminococcaceae_unclassified*	2.34^ab^	2.63^a^	1.86^bc^	1.57^c^	1.36^c^	0.127	<0.001	<0.001	<0.001
*Subdoligranulum*	3.40^b^	5.47^a^	3.14^b^	3.16^b^	1.78^c^	0.303	<0.001	0.006	0.002
*Lachnospiraceae_NK4A136_group*	1.27^b^	1.78^a^	0.74^c^	1.31^b^	0.50^c^	0.117	<0.001	0.010	0.019
*Alistipes*	1.32^b^	1.71^b^	0.81^c^	4.84^a^	0.78^c^	0.353	<0.001	0.423	0.302
*Christensenellaceae_R-7_group*	14.25^a^	14.20^a^	9.11^b^	8.68^b^	13.49^a^	0.643	0.007	0.124	0.003
*Firmicutes_unclassified*	2.98	3.18	2.93	3.23	2.84	0.115	0.798	0.788	0.789
*Lachnospiraceae_unclassified*	1.94	1.61	1.60	2.17	1.59	0.092	0.815	0.834	0.975

^a, b, c^Different superscript letters within a row indicate significant differences at *P* < 0.05. T1, NFC/NDF = 0.7; T2, NFC/NDF = 1.0; T3, NFC/NDF = 1.3; T4, NFC/NDF = 1.6; T5, NFC/NDF = 1.9.

#### 3.6.3. LDA effect size

**Figure 5:**
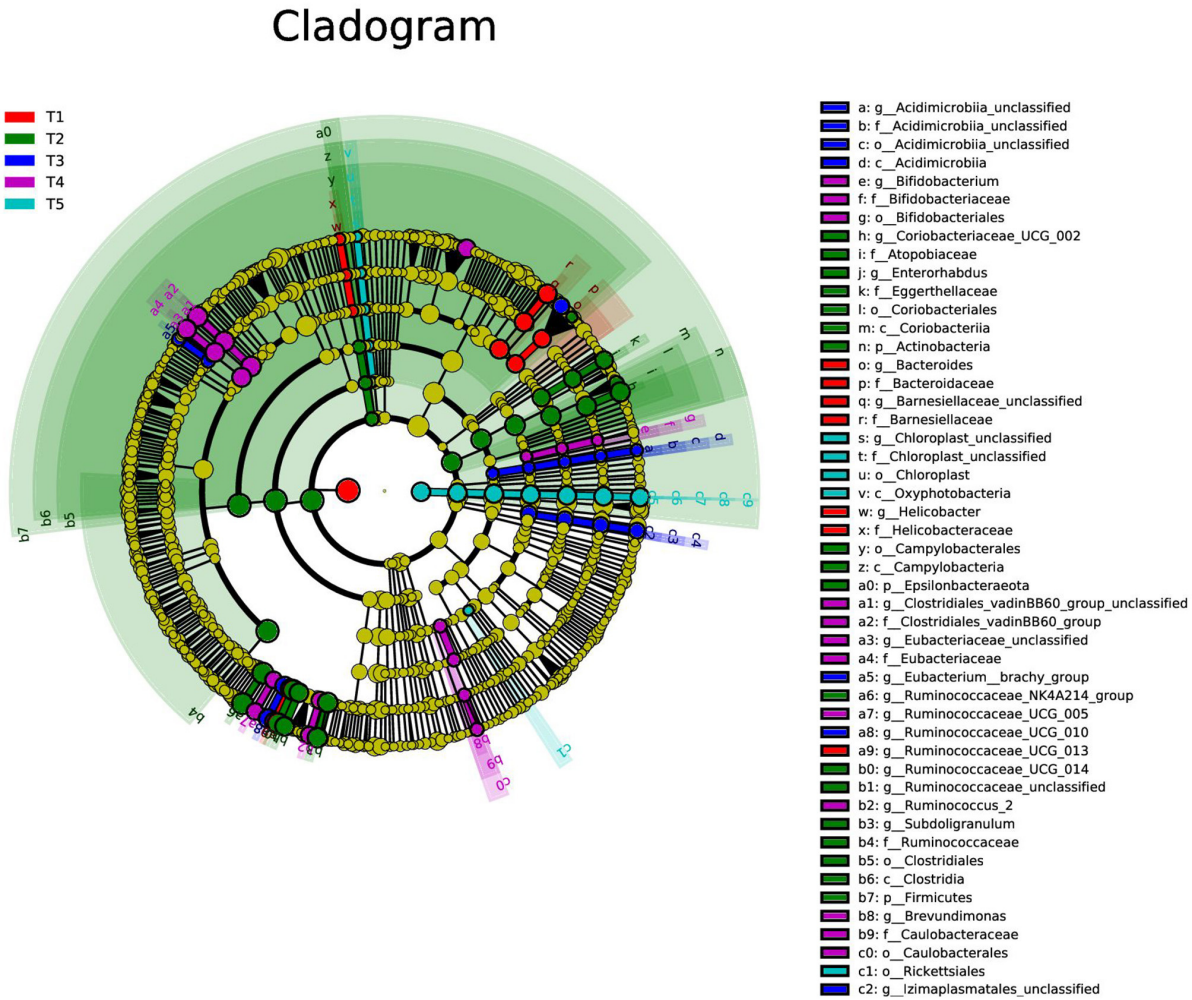
Cladogram of LEfSe multi-level species difference discriminant analysis (LDA > 2), different color nodes indicate microbial communities that are significantly enriched in the corresponding groups and significantly different between groups. Data are indicated as means ± SEM (*n* = 6). T1, NFC/NDF = 0.7; T2, NFC/NDF = 1.0; T3, NFC/NDF = 1.3; T4, NFC/NDF = 1.6; T5, NFC/NDF = 1.9.

The LDA effect size (LEfSe) was mainly used to obtain the final differential species by comparing between the groups. As shown in Figure 5, the most differential groups of cecum microorganisms were found in T2 rabbits and the least in T5 rabbits. A total of nine differential bacterial groups were screened in T2 rabbits for promoting intestinal health and cecum fermentation performance, namely, *Clostridiales, Clostridia, Ruminococcaceae, Ruminococcaceae_NK4A214_group*, and *Ruminococcaceae_ UCG_014* in Firmicutes and *Coriobacteriales, Coriobacteria, Coriobacteriaceae_UCG_002*, and *Eggerthellaceae* in Actinobacteria. However, the harmful differential bacteria, *Rickettsiales* which is symbiotic with the host, are screened in T5 rabbits.

### 3.7. Correlation analysis of altered cecal bacteria with pH, NH3-N, and VFA

**Figure 6:**
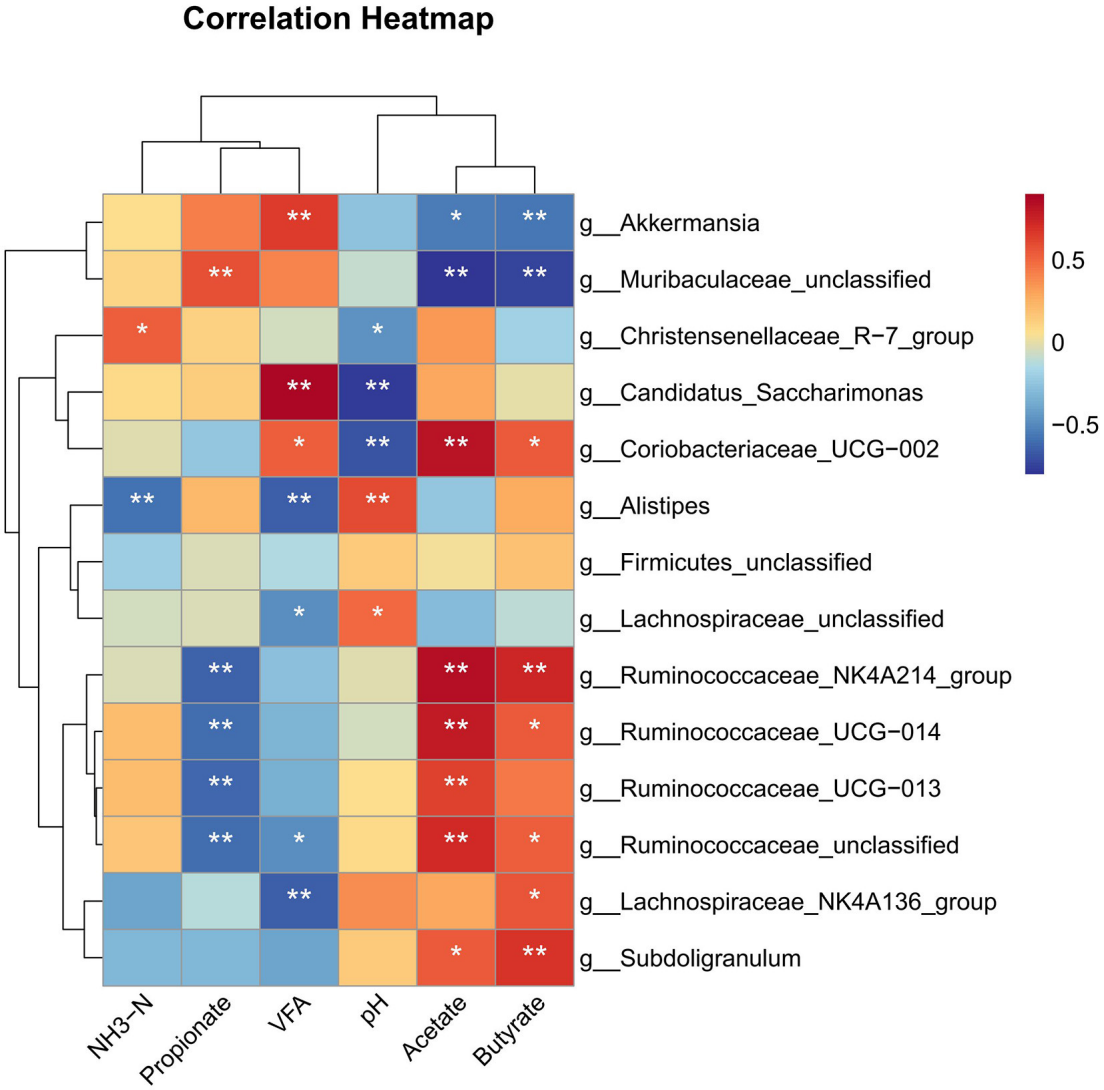
Spearman's rank correlation analysis (SRCA) between significantly modified microbiota, pH, NH_3_-N, and VFA concentrations of rabbits. *indicates *P* < 0.05 and **indicates *P* < 0.01. Red and blue colors represent positive and negative correlations, respectively. T1, NFC/NDF = 0.7; T2, NFC/NDF = 1.0; T3, NFC/NDF = 1.3; T4, NFC/NDF = 1.6; T5, NFC/NDF = 1.9.

Correlation analysis revealed that the burden of genus *Ruminococcaceae_NK4A214_group, Ruminococcaceae_UCG*−*014, and Ruminococcaceae_UCG*−*013* was positively correlated with acetate and negatively correlated with propionate (*P* < 0.01) (Figure 6). *Lachnospiraceae_NK4A136_group* showed a strong positive correlation with butyrate and a negative correlation with VFA (*P* < 0.05). The genus *Subdoligranulum* showed a positive correlation with acetate and butyrate (*P* < 0.05). The abundance of genus *Lachnospiraceae_unclassified* and *Alistipes* was positively correlated with pH and negatively correlated with VFA (*P* < 0.05); moreover, the relative burden of *Alistipes* was negatively correlated with NH_3_-N (*P* < 0.01). *Coriobacteriaceae_UCG*−*002* showed a positive correlation with VFA, acetate, and butyrate and negatively correlated with pH (*P* < 0.05). The abundance of genus *Candidatus_Saccharimonas* was positively correlated with VFA and negatively correlated with pH (*P* < 0.01). In addition, *Muribaculaceae_unclassified* and *Akkermansia* were negatively correlated with acetate and butyrate (*P* < 0.05).

## 4. Discussion

An appropriate level of dietary fiber is essential for animal growth and development, and excess or deficiency of fiber would limit the digestion and absorption of nutrients. We found that the ADG and ADFI of rabbits at NFC/NDF ratio = 0.7~1.3 were higher than those at NFC/NDF = 1.6–1.9, but the F/G, diarrhea rate, and mortality were lower than those at NFC/NDF = 1.6–1.9. A higher concentration of dietary fiber results in lower concentrations of other nutrients, and a compensatory increase in appetite as feedback which will lead to a higher F/G ratio. Meanwhile, the fiber can provide substrate for intestinal fermentation, produce VFA, maintain intestinal pH balance, and ensure normal activities of intestinal microorganisms; beneficial intestinal microorganisms and their fermentation products acting on antigen cells can improve the expression of immunoglobulins and macrophages in the body, thus regulating the immune function of the intestine, and nutritional diarrhea will be relieved. Moreover, NFC contains plant cell contents and the readily fermentable part, which will stimulate normal intestinal peristalsis, making the animals feel full. When dietary NFC is higher, the ADFI of rabbits is generally lower (Ranathunga et al., 2010; Chen et al., 2022).

**Figure 7:**
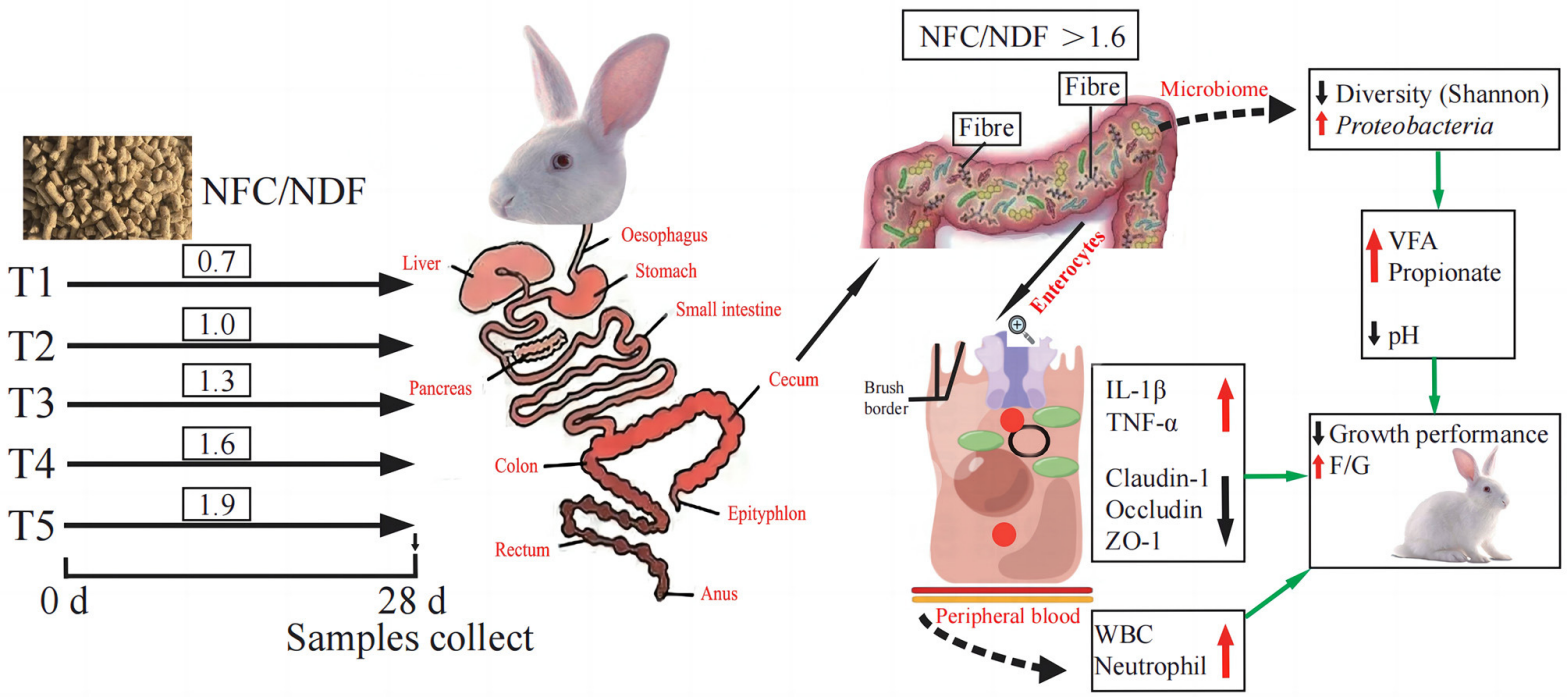
Schematic summary: effect of different ratios of dietary NFC/NDF on intestinal immunity, microbiota, and metabolites in rabbits.

Interleukins (IL) play an important role in immune cell activation and differentiation. However, high levels of IL may also induce an inflammatory response (Zhang et al., 2022). Il-1β and TNF-α are both pro-inflammatory cytokines, and TNF-α can promote the secretion of IL-1β (Torp et al., 2021). Immune diseases are often treated by blocking the expression of IL-1β clinically (Migliorini et al., 2020). Occludin, Claudins, and ZOs are the most important classes of genes related to intestinal immunity that play important roles in maintaining cell morphology and forming intestinal immunity (Wu et al., 2020; Tang et al., 2021). The expression of these genes will affect the function of the intestinal immunity and barrier. A summary of the main findings of this study is presented in Figure 7, and our results concluded that the expression of IL-1β and TNF-α genes induced by NFC/NDF = 0.7–1.3 was lower than that in NFC/NDF = 1.6–1.9. In parallel, the expression of Claudin-1, Occludin, and ZO-1 was higher at NFC/NDF = 1.0 and 1.3 than those at NFC/NDF = 1.6–1.9. It was found that appropriate levels of dietary fiber increased the numbers of lymphocytes in the rat (Fåk et al., 2015) and upregulated the gene expression of the anti-inflammatory cytokine IL-10 in the jejunum of a mouse, while downregulated the gene expression of the pro-inflammatory cytokines IL-1β, IL-6, and TNF-α (Xu et al., 2021). Moreover, appropriate levels of dietary fiber can increase the gene expression of Claudin-1, Occludin, and ZO-1 in the animal intestine (Chen et al., 2019; Beisner et al., 2021; Feng et al., 2022). Therefore, it was believed that intestinal immunity is better at NFC/NDF = 1.0 to 1.3 than that at NFC/NDF = 1.6 to 1.9. This may be due to the fact that dietary fiber can be fermented in the cecum to produce VFA, and meanwhile, it can increase the frequency of intestinal peristalsis and stimulate intestinal epithelial cells to secrete mucus (Mcrorie et al., 2020). Acetate and propionate can induce intestinal goblet cell differentiation and promote mucin synthesis (Yang et al., 2022). Acetate can also counteract TNF-α-induced epithelial barrier damage by regulating the expression of genes involved in the anti-inflammatory response in the intestine (Bach Knudsen et al., 2018; Al-Sadi et al., 2021) and reducing the transfer of virulence factors into the circulatory system (Aw and Fukuda, 2019). In addition, butyrate is the major energy source of intestinal epithelial cells, and it can effectively regulate the expression of intestinal immune factors, reduce intestinal permeability, and benefit intestinal health (Lenoir et al., 2020).

The organic acids regulate the macrophage activities in the intestine and may contribute to intestinal immune and barrier functions (Nouri, 2022). The cecum microorganisms of rabbits can produce VFA by fermenting cellulose and other components (Makki et al., 2018), and then, VFA can be quickly absorbed by the hindgut, providing 40% of the maintenance energy for rabbits (Read et al., 2019). The concentration of VFA in the cecum is an important indicator of the fermentation activity of cecum microorganisms. As presented in Figure 7, in this study, the pH value and acetate in the cecum were lowest at NFC/NDF = 1.9, and the VFA and propionate were highest at NFC/NDF ratio = 1.9. It has been proven in ruminants that excessive organic acids can lead to the occurrence of rumen acidosis, and in this study, we also found that a high NFC ratio can lead to a decrease in the pH of the cecum. Decreasing the dietary NDF can increase the concentration of VFA in the cecum of rabbits, decrease the pH of the cecum, and improve the fermentation pattern of the cecum (Wu et al., 2019; Wang et al., 2022a). Therefore, it is considered that the lower the NFC/NDF in the diet, the higher the fiber contents in the diet, the less the fermentable substances, and the poorer the ability of intestinal fermentation to produce acids, resulting in an increase in the pH of the cecum. However, diets with high levels of NFC/NDF ratio can provide the body with more fermentable carbohydrates, which can be fully fermented in the cecum to produce organic acids. In this case, the content of fiber is reduced, the digestion time is shortened, and the secretion of digestive juices is reduced, thus lowering the pH of the intestine (Gidenne, 2015).

The cecal microbiota is formed in the long-term evolution process and maintains a relatively stable balance with the host (Wassie et al., 2022). The balanced symbiosis between the host and microbiota can maintain animal health and improve performance (Halliday et al., 2019). In this study, according to the Alpha and Beta diversity results, there were significant differences in the cecal microbial diversity of rabbits in each group, indicating that different dietary NFC/NDF ratios would change the cecal microbiota diversity and richness. Both the diversity and richness of microorganisms at the NFC/NDF ratio = 1.9 were lower than other groups. Most studies have shown that animals fed a high-fiber diet have richer gut microbiota than those fed a low-fiber diet (Trompette et al., 2014). The addition of soluble fiber to the diet causes more microbial fermentation, which helps control the colonization of foodborne pathogens and reduces the incidence of diarrhea (Li et al., 2018). Moreover, diets with low fiber can lead to a decrease in the proportion of beneficial bacteria in the intestine (Simpson and Campbell, 2015). The comprehensive analysis shows that different dietary NFC/NDF ratios could affect the cecal microbial diversity of rabbits and change the dynamic balance of cecal microorganisms. The higher the ratio is, the lower the bacterial diversity and richness are. A possible explanation was that with the rising NFC/NDF ratio, NFC will be over-fermented in the cecum and lower the pH value of the cecum, which eventually leads to inhibit the growth of beneficial bacteria that cannot tolerate acidic environments and promote the reproduction of harmful bacteria.

The composition of dietary can affect the composition of the intestinal microbiota in rabbits, and dietary fiber plays an important role in the structure of rabbit microbiota (Pi et al., 2021). Previous studies showed that dietary fiber can improve intestinal function and microbial composition (Sadeghi et al., 2015). This experiment showed that when NFC/NDF ratio = 1.0–1.3, Firmicutes had the higher relative burden and Bacteroidetes had a lower relative burden than that of the other three groups. Firmicutes and Bacteroidetes are the main components of intestinal symbionts (Monteils et al., 2008; Hills et al., 2019), both of which can ferment dietary fat and fiber to produce short-chain fatty acids (Turnbaugh et al., 2008; Wang et al., 2022b). Firmicutes are involved in the absorption of protein and carbohydrates and contain a large number of fiber-degrading bacteria (Holscher, 2017). Bacteroidetes can promote carbohydrate fermentation (Gidenne, 2015). When the dietary NFC/NDF ratio is low, the fiber can be fully fermented in the cecum, which is conducive to the growth and reproduction of cellulolytic bacteria, thereby improving the conversion efficiency of dietary fiber (Ma et al., 2015). Therefore, it is believed that when the dietary NFC/NDF ratio = 1.0–1.3, the environment in the cecum of the rabbits is more conducive to the reproduction of beneficial bacteria and can effectively degrade dietary fiber. The results of the genus level also proved that the NDF with a dietary NFC/NDF ratio = 1.0–1.3 can be fully fermented in the cecum to produce abundant short-chain fatty acids, and then provide a beneficial physiological environment for the cecal microbiota. It is conducive to the survival of beneficial microorganisms such as *Ruminococcaceae, Lachnospiraceae*, and *Coriobacteriaceae* in the cecum, which has a positive effect on the balance of intestinal flora. In addition, we found that when the NFC/NDF ratio = 1.9, the relative burden of *Proteobacteria* was higher than that of other groups, and the relative burden of Actinobacteria was lower than that of other groups. It has been reported that Proteobacteria, including *Escherichia coli* and *Salmonella*, can easily cause intestinal inflammation in animals, followed by diarrhea (Shin et al., 2015). Actinobacteria mainly exist in the digestive of herbivores and can promote digestion (Chen et al., 2016). This experiment shows that when the dietary NFC/NDF ratio = 1.9, the burden of harmful bacteria in the intestine will increase, which is a serious threat to intestinal health.

We found that the pH value and acetate concentration in the cecum of rabbits decreased, while the propionate concentration increased with the increase in the ratio, as the dietary NFC/NDF increased. *Ruminococcaceae*, one of the most abundant genera of Firmicutes, which is also a producer of VFA, can effectively degrade fibers (Holscher, 2017; Dou et al., 2022). The results showed that the relative burden of *Ruminococcaceae_NK4A214_group, Ruminococcaceae_UCG-014*, and *Ruminococcaceae_UCG-013* was positively correlated with the concentration of acetate but negatively correlated with the concentration of propionate. It shows that the high dietary NFC/NDF ratio leads to the reduction of the relative abundance of *Ruminococcaceae*, the weakened ability to degrade fiber, and the reduction of the ability to produce acetate (Morrison and Miron, 2000). The *Christensenellaceae_R-7_group* is present in the gut microbiota of healthy hosts (Li et al., 2020). The results of this experiment showed that the relative burden of the *Christensenellaceae_R-7_group* was positively correlated with acetate and NH_3_-N. The *Christensenellaceae_R-7_group* belongs to Firmicutes and can improve the degradation of structural polysaccharides in the rumen. The genome of Firmicutes can encode glycoside hydrolase genes that degrade hemicellulose (Solden et al., 2018), and then maintain the health of the host by regulating the homeostasis of the intestinal environment. It is a short-chain fatty acid-producing bacteria that resist the invasion of pathogenic bacteria. Elevated acetate concentration causes an increased relative burden of the *Christensenellaceae_R-7_group* (Li et al., 2020). Previous studies have found that intestinal flora can provide nutrition and energy to the host by metabolizing carbohydrates and that *Lachnospiraceae* can break down straight-chain starch and the alpha-1,4 glycosidic bond in straight-chain starch to help animals utilize polysaccharides and fibrous material (Van Treuren and Dodd, 2020). This study showed that the relative burden of the *Lachnospiraceae_NK4A136_group* was positively correlated with butyrate (Cockburn et al., 2018). In addition, studies have confirmed that *Lachnospiraceae* can participate in the fermentation of fibrous substances or polysaccharides, and the main products are VFA such as butyric acid (Vacca et al., 2020), which is consistent with our results. From this, we conclude that cecal metabolites are determined by the degradation of fibrous and starch, and are positively associated with fermentation-related phenotypes and the burden of Firmicutes (Li et al., 2020).

## 5. Conclusion

Different dietary NFC/NDF can affect intestinal immune responses and the growth performance of rabbits. When dietary NFC/NDF = 1.0–1.3, the relative burden of Firmicutes was higher, which is more conducive to the decomposition of dietary fiber and the production of VFA. Dietary NFC/NDF = 1.0–1.3 can also improve the cecum microbial diversity of rabbits, which has a positive effect on the balance of intestinal flora. Importantly, it was also found that the cecal metabolites were determined by the degradation of fibrous and starch, which were positively associated with fermentation-related phenotypes and the burden of Firmicutes. Collectively, the mechanism may be associated with the appropriate NFC/NDF ratio, improving metabolites parameters by modulating gut microbiota.

## Data availability statement

The datasets presented in this study can be found in online repositories. The names of the repository/repositories and accession number(s) can be found below: NCBI Sequence Read Archive (SRA) database, https://www.ncbi.nlm.nih.gov/sra, PRJNA913458.

## Ethics statement

The animal study was reviewed and approved by all experimental procedures were approved by the Ethics Committee of Animal Experimentation of Hebei Agricultural University (Protocol 2021083).

## Author contributions

SL, TL, and BC: conceptualization. SL, TL, and KW: methodology, investigation, software, data curation, and writing—original draft preparation. KW and CL: validation and resources. TL and CL: formal analysis. XC and BC: writing review, editing, and supervision. BC: project administration and funding acquisition. All authors have agreed to the final manuscript.
