# Supplementary material for: The ratios of dietary non-fibrous carbohydrate (NFC) to neutral detergent fiber (NDF) influence intestinal immunity of rabbits by regulating gut microbiota composition and metabolites
Source: Front Microbiol. 2023 Apr 20;14:1146787. doi: 10.3389/fmicb.2023.1146787 (PMC10157162; doi:10.3389/fmicb.2023.1146787)
Supplement: Supplementary file 1 [file Data_Sheet_1.docx]

Supplementary Material

The Ratios of Dietary Non-Fibrous Carbohydrate (NFC) to Neutral Detergent Fiber (NDF) Influence Intestinal Immunity of Rabbits by regulating Gut Microbiota Composition and Metabolites

Shuo Li^1†^, Tingting Liu^2,3†^, Kun Wang^3†^, Chong Li^4,5^, Fengyang Wu^2,6^, Xinyu Yang^2^, Man Zhao^2^, Xiang Chen^1*^ and Baojiang Chen^2*^

^1^ College of Animal Science, Guizhou University, Guiyang 550025, China

^2^ College of Animal Science and Technology, Hebei Agricultural University, Baoding 071000, China

^3^ Institute of Cereal and Oil Crops, Hebei Academy of Agriculture and Forestry Sciences, Shijiazhuang 050035, China

^4^ Key Laboratory for Feed Biotechnology of the Ministry of Agriculture and Rural Affairs, Institute of Feed Research, Chinese Academy of Agriculture Sciences, Beijing 100081, China

^5^ Precision Livestock and Nutrition Laboratory, Teaching and Research Centre (TERRA), Gembloux Agro-Bio Tech, University of Liège, Gembloux 5030, Belgium

^6^ College of Food Science and Technology, Hebei Agricultural University, Baoding 071000, China

^†^ These authors have contributed equally to this work

*** Correspondence:**Baojiang Chen E-mail: chenbaojiang@vip.sina.com

**Additional file 1:**

**Supplementary Table S1.** Chemical composition levels of basal diets (air-dried basis) %.

| Ingredients | T1 | T2 | T3 | T4 | T5 |
| --- | --- | --- | --- | --- | --- |
| Corn | 0.00 | 8.50 | 16.50 | 25.00 | 33.60 |
| Wheat bran | 11.00 | 11.00 | 10.00 | 11.00 | 11.50 |
| Soybean meal | 15.00 | 15.50 | 16.00 | 16.00 | 16.00 |
| Alfalfa meal | 26.50 | 26.00 | 26.00 | 25.50 | 25.50 |
| Oat meal | 43.50 | 35.00 | 27.50 | 18.50 | 9.40 |
| NaCl | 0.50 | 0.50 | 0.50 | 0.50 | 0.50 |
| Lys | 0.08 | 0.08 | 0.08 | 0.08 | 0.08 |
| Met | 0.12 | 0.12 | 0.12 | 0.12 | 0.12 |
| Zeolite meal | 1.00 | 1.00 | 1.00 | 1.00 | 1.00 |
| Premix^a^ | 0.30 | 0.30 | 0.30 | 0.30 | 0.30 |
| Palm oil meal | 1.00 | 0.80 | 0.50 | 0.20 | 0.00 |
| Bentonite | 1.00 | 1.20 | 1.50 | 1.80 | 2.00 |
| Total | 100.00 | 100.00 | 100.00 | 100.00 | 100.00 |
| Nutrient levels^b^ | | |  |  |  |
| DM | 89.21 | 89.43 | 89.01 | 88.95 | 88.90 |
| CP | 17.11 | 17.13 | 17.14 | 17.11 | 17.12 |
| EE | 3.23 | 3.29 | 3.21 | 3.21 | 3.28 |
| CF | 17.70 | 15.71 | 13.99 | 11.92 | 9.89 |
| NDF | 41.52 | 38.25 | 35.24 | 32.00 | 28.72 |
| ADF | 21.69 | 19.34 | 17.30 | 14.86 | 12.46 |
| NFC/NDF^c^ | 0.72 | 1.02 | 1.30 | 1.60 | 1.90 |

a. The premix provided per kg of the diet: Fe (as ferric sulfate) 70 mg, Cu (as copper sulfate) 20 mg, Zn (as zinc sulfate) 70 mg, Mn (as manganese sulfate) 10 mg, Se (as sodium sulfate) 0.25 mg, Co 0.15 mg, I 0.2 mg, VA 10 000 IU, VD 900 IU, VE 50 mg, VK 2 mg, VB_1_ 2 mg, VB_2_ 6 mg, VB_12_ 0.02 mg, Pantothenic acid 50 mg, Pyridoxine 2 mg, Nicotinic acid 50 mg, Choline 1 000 mg, Biotin 0.2 mg.

b. Nutrient levels are measured values.

c. Non-fibrous carbohydrates, including all cell contents and the pectin galactose component in the cell wall.

**Additional file 2:**

# Materials and Methods:

**Cecal content DNA Extraction, 16S rRNA Amplification and Sequencing**

Six cecal content samples in each group were randomly selected and used for analysis of the intestinal microbiota. Following manufacturer's protocol, the Bacterial Genomic DNA (gDNA) was extracted from samples using Power Soil DNA Isolation Kit (Omega Bio-Tek Inc., Norcross, GA, USA). The quality and quantity of DNA were assessed by 260 nm/280 nm and 260 nm/230 nm ratios and stored at -80°C till further analysis.

Through polymerase chain reaction (PCR), the V3-V4 bacterial region of 16S rRNA gene was amplified using 338-F (5' ACTCCTACGGAGG CAGCA -3') and 806-R (5'-GGACTACHVGGGTWTCTAAT-3') primers combined with adapter and barcode sequences. A total of 50 μL PCR mixture containing buffer and high GC enhancer (10 μL each), Q5 high-fidelity DNA polymerase (0.2 μL), dNTP (1 μL), primer (10 μM each) and gDNA (60 ng) were prepared. The PCR was performed as: an initial denaturation (95°C for 5 min), followed by 15 cycles (95°C for 1 min, 50°C for 1 min and 72°C for 1 min) and final extension (72°C for 7 min). These first step PCR products were purified using VAHTS™ DNA Clean Beads (Vazyme Biotech Co., Ltd., China). Then the second step of PCR was performed with a 40 μL PCR mixture containing 2× Phμsion HF MM (20 μL), ddH2O (8 μL), primer (10 μM each) and first step PCR products (10 μL). The PCR conditions were as follows: an initial denaturation (98°C for 30 s), followed by 10 cycles (98°C for 10 s, 65°C for 30 s and 72°C for 30 s) and final extension (72°C for 5 min). All PCR products were quantified by Quant-iT™ dsDNA HS reagent (Calbiochem Co., Ltd., Germany) and pooled together. High-throughput sequencing analysis of bacterial rRNA gene was done using the Illumina Hiseq 2500 platform (2 × 250 paired ends) at Biomarker Technologies Corporation, Beijing, China.

According to the relationship between paired-end (PE) reads and overlapping reads, the double-ended sequencing data was compiled into a sequence of tags after Hiseq sequencing. The quality of the reads and the effect of merging analyzed by quality control were used to obtain valid data by three steps method (including PE read splicing, tag filtering and the removal of chimerism). UCLUST was used in QIIME (version 1.8.0) software to cluster tags at a similarity level of 97% to obtain operational taxon unit (OTUs) and classify the OTUs based on Silva taxonomy database (<https://www.arb-silva.de/>).
